# Psychologists’ experiences towards culturally responsive practices to strengthen social and emotional wellbeing with Aboriginal and Torres Strait Islander clients

**DOI:** 10.1080/00049530.2024.2356116

**Published:** 2024-05-27

**Authors:** Emily Darnett, Andrew Peters, Monica Thielking

**Affiliations:** a Department of Psychological Sciences, Swinburne University of Technology, Melbourne, Australia; b The Westerman Jilya Institute for Indigenous Mental Health, Melbourne, Australia

**Keywords:** Indigenous psychology, social and emotional wellbeing, mental health

## Abstract

**Objective:**

This study aimed to explore psychologists experiences when providing culturally responsive psychological practice working with Aboriginal and Torres Strait Islander^1^ clients.

**Method:**

Psychologists (*N* = 108, Female 83.2%, Male 16.8%, Aboriginal 13.9%, non-Indigenous 86.1%, age range 22–83) responded to an electronic mixed method survey. Statistical and content analysis were undertaken using data gathered to address the study aims.

**Results:**

The findings highlighted and validated challenging, successful, and unsuccessful psychological practice adjustments used by psychologists when working with Aboriginal and Torres Strait Islander clients. Aboriginal psychologist’s voices were privileged as traditional knowledge holders.

**Conclusions:**

There is diversity in opinion and practices utilised by psychologists when working with Aboriginal and Torres Strait Islander peoples. The uncertainty applied to the effectiveness of treatments, and/or outcomes. To enhance social and emotional wellbeing higher education institutes need to embed Aboriginal and Torres Strait Islander content in psychology curriculum to better prepare non-Indigenous psychologists to work in a culturally responsive way with Aboriginal and Torres Strait Islander clients.

## Key Points

**What is already known about this topic**:

1. Aboriginal and Torres Strait Islander people’s worldviews differ from Western worldviews. These worldviews shape how mental health and wellbeing are explained, enhanced, and maintained.
2. Psychology training perpetuates Westernised viewpoints with little education about Aboriginal and Torres Strait Islander wellbeing.
3. The majority of mainstream psychological services are currently delivered by non-Indigenous psychologists.

**What this topic adds**:

1. Articulates challenges experienced by Aboriginal and non-Indigenous psychologists when working with Aboriginal and Torres Strait Islander clients.
2. Emphasises successful and unsuccessful clinical and cultural psychological practices for psychologists to consider when working with Aboriginal and Torres Strait Islander clients.
3. Highlights the need for reduced systemic barriers, and improved psychology training in higher education.

Caveat

The research team would like to note that due to the nature of qualitative analysis, some examples included in the findings may be upsetting to Aboriginal and Torres Strait Islander readers. To report the findings accurately, the participants’ qualitative responses were presented as verbatim as possible, which at times included their bias.

## Introduction

This paper reports the findings from original research that explored culturally responsive practices by psychologists when working with Aboriginal and Torres Strait Islander peoples of Australia. The term Aboriginal and Torres Strait Islander is respectfully used throughout this article to refer to both Indigenous groups of Australia. Aboriginal and Torres Strait Islander peoples are often grouped together in psychology research due to similarities in cultures and experiences (e.g., Clark & Hirvonen, 2022; Dawson et al., 2023; Dudgeon & Kelly, 2014; Dudgeon et al., 2014; Gaborit et al., 2022). It is important to recognise the diversity in cultures, customs, traditions, ceremonies and experiences between Aboriginal peoples of Australia and the Torres Strait Islander peoples of the Torres Strait Islands, but also the diversity between different Aboriginal groups within Australia. When the term Aboriginal is used in this paper, it only refers to specific participants and/or literature that have self-reported to be Aboriginal or relate to Aboriginal experiences and knowledge. In all aspects, the authors have aimed to respect the diversity between Aboriginal and Torres Strait Islander cultures. This paper aims to provide non-Indigenous psychologists with a set of recommendations on culturally responsive psychological practices that are derived from the findings which they can develop in their work to enhance Aboriginal clients’ social and emotional wellbeing (SEWB).

Many terms have been used in past literature to describe the process of embedding culturally responsive practice into mental health service provision, including cultural competency, cultural safety, cultural awareness, cultural sensitivity, and cultural responsiveness (Curtis et al., 2019; Dudgeon & Walker, 2015; Dudgeon et al., 2014; Green et al., 2016; Kilcullen et al., 2018; J. O’connor et al., 2015; Smith et al., 2021; Westerman & Dear, 2023). Each of these terms has been critiqued for being too static, unattainable, broad, and insufficient in their descriptions or encumberment of cultural humility (Curtis et al., 2019; Smith et al., 2021). However, cultural responsiveness has emerged in such debates to be the most appropriate term as it has evolved to encompass competence within practice, as well as critical reflection, and relationships that are collaborative and respectful (Green et al., 2016; Smith et al., 2021). It is for this reason that cultural responsiveness will be used throughout this paper unless another term is used in the research or commentary being cited.

Culture plays a leading role in the wellbeing of Aboriginal and Torres Strait Islander peoples (Dudgeon & Walker, 2015; Dudgeon et al., 2014; Gee et al., 2014; Gee et al., 2023; Shepherd, 2019). The disruption to cultural continuation that colonisation caused has resulted in Aboriginal and Torres Strait Islander peoples experiencing detrimental impacts on SEWB (Dudgeon & Walker, 2015; Dudgeon et al., 2014; Gee et al., 2014; Gee et al., 2023). However, most mental health services and psychologists are non-Indigenous, working in mainstream settings such as hospitals or schools, and practicing through a Westernised lens (Clark & Hirvonen, 2022; Dudgeon & Walker, 2015; Westerman, 2004, 2008, 2010; Westerman & Dear, 2023). With culture being a protective factor for many Aboriginal and Torres Strait Islander peoples, psychologists need to practice in a culturally responsive way. Psychologist should know how and when to appropriately incorporate culture into the Westernised psychology practices in order to be an effective source of support for Aboriginal and Torres Strait Islander peoples (Clark & Hirvonen, 2022; Dudgeon et al., 2014; Geerlings et al., 2018; Smith et al., 2021; Westerman, 2004, 2010).

The ability to competently provide psychological services with clients from diverse cultural backgrounds is one of the eight core professional competencies of graduating psychologists imposed by the Psychology Board of Australia (AHPRA, 2017). This is particularly important given Australia’s burgeoning multicultural population that increased by almost one million immigrants over 5 years (ABS, 2022). Whilst cultural considerations are mentioned throughout higher education psychology courses (Geerlings et al., 2018; Gillan et al., 2017), the preference for Westernised practice models and frameworks is not fully effective in equipping students to develop the necessary skills to work with Aboriginal and Torres Strait Islander populations (Dudgeon et al., 2014; Gee et al., 2014; Geerlings et al., 2018; Mcconnochie et al., 2012; Mullins & Khawaja, 2018; Westerman & Dear, 2023). Moreover, often psychology students will not encounter an Aboriginal and Torres Strait Islander client until after they have completed their training further limiting their exposure and ability to develop culturally responsive practices. Furthermore, there are limited empirical-based interventions and resources available to guide non-Indigenous psychologists to decolonise their practice when working with Aboriginal and Torres Strait Islander clients.

Mcconnochie et al. (2012) held structured interviews with 23 non-Indigenous psychologists to explore how they adopted culturally appropriate strategies when working with Aboriginal and Torres Strait Islander clients. The findings suggested that the non-Indigenous psychologists were undertaking a “trial and error” approach to working with Aboriginal and Torres Strait Islander clients as they did not gain the necessary education during their formalised training (Mcconnochie et al., 2012). For an evidence-based profession that prides itself on the scientific-practitioner model of practice, this approach is questionable. It is not aligned with the underlying tenets of the psychological professional which is based on the principle of “do no harm”. This method is likely to have perpetuated further harm to the Aboriginal and Torres Strait Islander clients who were the “trial” clients, and consequently, ended in “error”. This may result in the compounding of negative experiences accumulated through years of previous engagement with Westernised systems, like mental health services. These negative experiences can have greater impacts on Aboriginal and Torres Strait Islander peoples due to the residual and ongoing impacts of colonisation (Clark & Hirvonen, 2022). Overall, there are a range of unique challenges that occur for Aboriginal and Torres Strait Islander peoples that span historical, political, and social landscapes, which need to be known and constantly considered by non-Indigenous psychologists.

It is important to also explore the challenges and barriers that exist for non-Indigenous psychologists working with Aboriginal and Torres Strait Islander clients. Firstly, non-Indigenous psychologists may possess limited understanding in relation to the broad range of historical and current factors that continue to influence the health and wellbeing of Aboriginal and Torres Strait Islander peoples (Westerman, 2010). Further, it has been suggested that non-Indigenous psychologists often lack knowledge of Indigenous customs, and norms, often resulting in a weak rapport being established between therapist and clients (Westerman, 2010). Moreover, it has been suggested that misdiagnosis has occurred, due to non-Indigenous psychologists, having an inaccurate understanding of culture, community and context (Dudgeon et al., 2014; Westerman, 2004, 2010; Westerman & Dear, 2023). Lastly, non-Indigenous psychologists’ worldviews likely differ from their Aboriginal and Torres Strait Islander clients (Dudgeon et al., 2014; Dudgeon & Walker, 2015; Gee et al., 2014; Westerman, 2010). Therefore, non-Indigenous psychologists may find the process of exploring these discrepancies between worldviews time consuming and challenging (Westerman, 2021). To aid the development of cultural responsivity in this area, Kilcullen et al. (2018) suggests psychologists need to develop awareness about their personal worldview and how their experiences differ from the challenges and barriers experienced by Aboriginal and Torres Strait Islander clients through critical reflective practices.

Past research has attempted to explore effective ways in which psychologists can provide culturally responsive practices while using established Westernised approaches when working with Aboriginal and Torres Strait Islander clients. For example, Ponturo and Kilcullen (2021) conducted a systematic review to find effective evidence-based psychological interventions employed with Aboriginal and Torres Strait Islander clients. Qualitative synthesis of the results from 12 included studies suggested that narrative therapy, Cognitive-Behavioural Therapy (CBT), acceptance-based therapies, and multisystemic therapy were reported to have cross-cultural applications (Ponturo & Kilcullen, 2021). However, given the diverse evaluation methods employed in the included studies, the qualitative synthesis could only speak to the cross-cultural applications of some elements of the therapies suggested, and not the therapies as a whole. They concluded that the suggested psychological interventions have the potential to enhance Aboriginal and Torres Strait Islander SEWB, however, more studies exploring interventions, and outcomes would be necessary to understand the applicability of the therapies.

Dudgeon and Kelly (2014) and Ponturo and Kilcullen’s (2021) agree CBT can be a useful tool when working with Aboriginal and Torres Strait Islander clients. However, Dudgeon and Kelly (2014) suggested CBT may be an inadequate tool when used in isolation. They posited that CBT when used alone is not culturally responsive as it was not developed based on Indigenous paradigms of wellbeing (Dudgeon & Kelly, 2014). Dudgeon and Kelly (2014) concluded by advocating for the need to consider interventions such as CBT to be part of the tools that are utilised within a broader framework that Aboriginal and Torres Strait Islander people require to strengthen SEWB. Whilst Dudgeon and Kelly’s (2014) commentary focused on psychological therapies such as CBT, their suggestion to consider most psychological therapies within a broader framework underpinned by Indigenous paradigms can be broadly applied.

There is considerable overlap within the literature on what approaches effectively contribute to culturally responsive psychological services with Aboriginal and Torres Strait Islander clients. Harnessing a culturally appropriate lens and using Indigenous frameworks when providing psychological services to Aboriginal and Torres Strait Islander clients is recommended throughout the literature (Bullen et al., 2023; Dawson et al., 2023; Dudgeon et al., 2014; Gee et al., 2014; Gee et al., 2023; Kilcullen et al., 2018; Krakouer et al., 2022; Murrup‐Stewart et al., 2019; Westerman 2004, 2008, 2010). Rhodes and Langtiw (2018) argued that psychology services need to utilise community‐based approaches to mental health. This recommendation has been supported in the literature with additional researchers adding suggestions that highlight the need for services to be relational, strengths-based, and participatory practices when supporting Aboriginal and Torres Strait Islander clients (Bullen et al., 2023; Clark & Hirvonen, 2022; Dawson et al., 2023; Dudgeon et al., 2014; Gee et al., 2014; Gee et al., 2023; Kilcullen et al., 2018; Krakouer et al., 2022; Mullins & Khawaja, 2018; Westerman & Sheridan, 2020).

Kilcullen et al. (2018), further expanded on this research, by conducting a qualitative study aimed at exploring 19 urban Aboriginal and Torres Strait Islander people’s perspectives on health and wellbeing to inform more culturally appropriate methods of health delivery. The findings supported holistic, values-based approaches enhanced mental health and wellbeing which is echoed repeatedly in the literature (Bullen et al., 2023; Dawson et al., 2023; Dudgeon et al., 2014; Gee et al., 2014; Krakouer et al., 2022; Murrup‐Stewart et al., 2019; J. O’connor et al., 2015). Moreover, Murrup‐Stewart et al. (2019) undertook a systematic review of 33 articles in order to contribute to the evidence-base supporting the need for Indigenous self-determination by analysing Aboriginal perspectives of SEWB programmes. The review found self-determined programmes to be more successful to increase Aboriginal peoples SEWB highlighting the importance of self-determination for Aboriginal and Torres Strait Islander peoples. Self-determination is arguably one of the most commonly occurring recommendations in previous research to enhance engagement (Clark & Hirvonen, 2022; Coombs, 2023; Dawson et al., 2023; Dudgeon et al., 2014; Gee et al., 2014; Mullins & Khawaja, 2018; Westerman & Sheridan, 2020). Importantly, Murrup‐Stewart et al. (2019) reported the quality of the 33 included articles to be the majority minimum standard (70%) and advocated for the closer adherence to NHMRC guidelines in future research.

Mullins and Khawaja (2018) interviewed 12 non-Indigenous psychologists to investigate their perspectives on how they enacted clinical and cultural competence when working with their Aboriginal and Torres Strait Islander clients. Their findings indicated that the most common first step to improve psychology practice reported by participants was to engage with the local community (Mullins & Khawaja, 2018). Whilst this body of literature is mostly cohesive in suggestions for culturally responsive approaches that improve clinical practices and programmes to increase SEWB, the research is often theoretical. A more in-depth understanding of what these approaches look like in practice, and studies of their effectiveness in improving Aboriginal and Torres Strait Islander mental health would be beneficial to both non-Indigenous and Indigenous psychologists’ practices.

In summary, many researchers have suggested different ways in which psychologists can improve their cultural responsivity in practice to strengthen Aboriginal and Torres Strait Islander people’s SEWB. This study aims to expand upon the previous research and further explore how psychologists believe they are providing culturally responsive practice when working with Aboriginal and Torres Strait Islander clients. It will add unique insights by investigating the perspectives of psychologists who both identify as Aboriginal as well as non-Indigenous, which has been a missing element in the body of literature to date. It is hoped that learnings from Aboriginal psychologists in relation to how they support communities will be shared and inform non-Indigenous psychologists’ cultural responsiveness.

## Method

### Research design

This study employed a mixed-method approach to explore psychologists’ experiences of working with Aboriginal and Torres Strait Islander clients to strengthen SEWB.

### Participant recruitment

The participants were recruited via their association with the Australian Psychological Society (APS), various social media platforms, and university affiliations through email invitations. To participate in the survey, people followed the Qualtrics link that was in the advertisement emails and poster.

To privilege Indigenous voices and perspectives in this study, group allocation was utilised. By separating Aboriginal participants responses from that of their non-Indigenous counterparts this study holds space for the Indigenous knowledges without distorting or whitewashing, this way we can continue to dismantle the colonial identities that have historically concealed our voices (Kunnie & Goduka, 2017). If the participants identified as Aboriginal, they were allocated to group 1. If participants indicated they were non-Indigenous and currently or have previously worked with Aboriginal and Torres Strait Islander clients they were allocated to group 2. Participants who identified as non-Indigenous and indicated they had not previously work as a psychologist with any Aboriginal and Torres Strait Islander clients before, were placed in group 3.

1. “How do you or the clinic/organisation you work for identify Aboriginal and/or Torres Strait Islander clients?”,
2. “Do you think psychologists need to adjust their psychological practice when working with Aboriginal and/or Torres Strait Islander clients? Please explain your answer”,
3. “During your tertiary education, do you feel you and your peers were provided with enough education to adequately adjust your psychological practice to work with Aboriginal and/or Torres Strait Islander clients? Please explain your answer”

Questionnaire. Regardless of the group allocation (e.g., group 1, 2, or 3), all participants’ demographic questions remained the same. For comparison purposes, the demographic questions aligned with the Australian Health Practitioner Regulation Agency (AHPRA) annual data collection survey that provides insights into the current demographic profile of the psychology workforce. Outside of demographic questions all participants were asked the same three questions that created the bases for the broader exploration into psychology practice and higher education. These included:

This study as previously stated was part of a broader investigation into psychology practice and education. This specific paper explored the psychological practice element. The education questions were reported in a forthcoming paper. Therefore, only the findings from the questions post-group allocation will be reported in this paper. Participants were asked to respond to questions that matched the group to which they belonged. Aboriginal participants in group 1 received questions that acknowledged their cultural knowledges. For example, Aboriginal psychologists in group 1 were asked

1. “Please explain any challenges if any, that you have experienced when working with Aboriginal and/or Torres Strait Islander client/s?”,
2. “What practice adjustments have you found to be successful when working with Aboriginal and/or Torres Strait Islander client/s?”,
3. “What practice adjustments, if any, have you found to not be successful when working with Aboriginal and/or Torres Strait Islander client/s?”.

There was no assumed Indigenous cultural understanding or knowledge reflected in the questions posed to group 2 or 3. Participants in group 2 held assumed clinical skills and therefore the questions posed to them were

1. “Please explain any challenges that you have faced when working with Aboriginal and/or Torres Strait Islander client/s?”,
2. “What practice adjustments have you found to be successful when working with Aboriginal and/or Torres Strait Islander client/s? Please explain your answer”,
3. “What practice adjustments have you found to not be successful when working with Aboriginal and/or Torres Strait Islander client/s? Please explain your answer”,
4. “Do you feel you have enough cultural awareness and knowledge to successfully adjust your practice for Aboriginal and/or Torres Strait Islander client/s? If so, please explain how you obtained the knowledge?”,
5. “Do you feel you know enough about the challenges facing Aboriginal and/or Torres Strait Islander peoples and communities to provide competent psychological therapy to an Aboriginal and/or Torres Strait Islander client/s? Please explain your answer”.

The participants in group 3 were not able to draw upon their clinical experiences to provide relevant answers regarding working with Aboriginal and Torres Strait Islander clients. Therefore, the majority of questions posed to group 3 aimed to explore participants’ satisfaction with the preparations to work with Aboriginal and Torres Strait Islander clients provided by higher education institutes. Originally the results from this study were planned to be presented in the same article, however, given the rich insights provided by all participants and publication limitations, the research team decided to divide the findings into 2 separate articles with more targeted focuses on practice, and education. Therefore, given group 3’s education focused questions, they will predominantly be reported in another paper with a higher education focus. The question posed and findings reported from group 3 that are explored in this paper include

1. “Do you feel you have enough knowledge about the Aboriginal and/or Torres Strait Islander culture to safely and successfully provide psychological support to Aboriginal and/or Torres Strait Islander clients?”

The inclusion of group 3’s data returned a large amount of robust and in-depth insights. These will likely provide useful insights into further reform of the psychology curriculum development and teaching practices towards a decolonised approach in higher educational institutes. Therefore, to give these findings the chance to make the most meaningful impact, they will be discussed in a follow-up article. This is also true for any of the education or training-related responses from the three groups.

### Data collection

The Qualtrics electronic survey was created with both open and closed questions to capture more robust qualitative and quantitative responses provided by participants. This allowed for the participants to elaborate upon their answers when they determined this to be useful to explain their experience. To collect the data an anonymous Qualtrics link was sent to participants via email and advertised on social media platforms inviting them to participate. Data was collated in Qualtrics and downloaded to an SPSS file to undertake quantitative data analysis.

### Data analysis

IBM statistical software package SPSS version 29.0.1.0 (171) was used to analyse the quantitative data collected from participants. As the demographic questions asked in the survey aligned with those asked by AHPRA on annual bases, categorical answers were not always the most appropriate to capture the complete demographics of the psychological workforce. To better reflect the complexities of participants identities and promote inclusion, often space was left for open-ended responses and “other” option with a textbox for entering data outside of the categorical responses (Hughes et al., 2016). This meant that the raw data collected in the responses to demographic questions that was not originally categorical was coded into a numerical categorical format for cross comparisons (Hughes et al., 2016).

The frequency and descriptive statistics were calculated to understand the categorical demographics of the participant sample. Chi-Squared Goodness of Fit tests were used to compare the demographic characteristics of the study participants with AHPRA survey data. This was done to make comparisons and gain insight into some limitations of the study. Categories were combined appropriately for categories with low counts.

Due to group imbalances resulting in some small sample sizes, exact hypothesis testing in the form of Fisher Freeman-Holton (FFH) test of significance was utilised to compare the demographic characteristics of the groups. FFH was used to explore the significance between three groups on the categorical variables. To allow for multiple testing only p-values below 0.005 were regarded as significant (Etymologia: Bonferroni correction. (2015), and Cramer’s V (*V*) was used to describe the strength of all associations (Akoglu, 2018).

All demographic questions were coded to be categorical except for three continuous variables. To compare the groups in terms of the continuous variables of age, hours participants spent working in the public and private sectors, and the number of years spent practicing as a psychologist, nonparametric Kruskal-Wallis tests were conducted allowing for any departures from a normal distribution.

To analyse the qualitative data collected from the open-ended questions in the survey an inductive approach to conventional content analysis was employed (Hsieh & Shannon, 2005; Vears & Gillam, 2022). As such, the codes used to label the data were developed during the process of coding. This data analysis methodology has been successfully used in research that impact Aboriginal and Torres Strait Islander peoples (Dawson et al., 2014; Gaborit et al., 2022). The Aboriginal first author analysed, categorised, and counted the themes that emerged acknowledging the Indigenous worldview that they inherently applied which grounded the Aboriginal psychologists’ knowledges in culture and presented them in context, as well as critically assess the construction of colonial knowledge about Aboriginal and Torres Strait Islander peoples (Ardill, 2013; Nakata, 2007). Approximately ten percent of data was cross coded by the non-Indigenous last author to ensure inter-rater reliability (O’Connor & Joffe, 2020). Given the anonymity of the survey in line with maintaining confidentiality for research participants, no member checking component was available to be undertaken.

Lastly, whilst the content analysis was underway, frequencies of reoccurring content was recorded (Singer & Couper, 2017). This allowed for a second quantitative data analysis that simply placed a numerical percentage to the main themes which indicated how regularly each theme was suggested by the participants. Importantly, the data collected from participants was analysed and reported according to group allocation when meaningful. This was undertaken to highlight and respect Aboriginal psychologists’ knowledge and encourage deep consideration and further exploration of the non-Indigenous participants suggestions.

### Ethics

Ethics approval was granted by Swinburne University’s Human Research Ethics Committee (ref: 20235840–14219). Further to align with Aboriginal and Torres Strait Islander ethical research the following points should be highlighted. This research was undertaken to respond to a priority determined by the majority Aboriginal research team through past research and lived experience. The research was led by an Aboriginal higher degree research student in partial fulfilment of a Doctor of Philosophy (Clinical Psychology) degree at Swinburne University. Further, the research team is majority Aboriginal. Outside of the research team, further Aboriginal and Torres Strait Islander governance was sought in an informal capacity through community connections and Indigenous colleagues.

Given the target population of this research being psychologists, an Indigenous methodology was not undertaken. The research was focused on their roles as psychologists, rather than their cultural backgrounds. Psychologists need a minimum level of higher education to satisfy the AHPRA registration board requirements. Therefore, working on this assumption, participants know how to navigate the Western higher education systems, and have a reasonably high level of literacy, numeracy, and computer skills. For these reasons, an electronic survey was the best method for data collection to balance time burden and the use of open-ended questions allowed for elaboration.

The research was guided by the SEWB framework commonly used to explain Aboriginal and Torres Strait Islander wellbeing (see Gee et al., 2014). This research takes a strengths-based approach in the methodology and manuscript to move beyond practices that have historically perpetuated harm to Aboriginal and Torres Strait Islander people. The research plans to translate these findings into sustainable curriculum and practice reform. Whilst the research may not have directly benefited the Aboriginal participants, the wider benefits of the Aboriginal and Torres Strait Islander communities are likely to far outweigh the time burden of participants. The research directly strengthened the first author’s capacities in the research industry, and through the informal governance yarns and the knowledge translation in this paper, there were many opportunities presented on a macro level for people to learn from one another.

## Results

In total, 115 people completed the online survey. Of those, seven were removed as their survey responses were less than 50% complete, leaving a sample of 108 participants. In this sample 89 described their gender as female, and 18 described their gender as male. Further, 15 participants (13.9%) identified as Aboriginal, and 93 participants (86.1%) identified as non-Indigenous. The age range across the sample was 22–83 years (*M = 40.7, SD = 12.84*). Table 1 provides an overview of the total demographics of participants in comparison to the data collected from AHPRA’s annual report 2022/23 (AHPRA, 2023).

**Table 1.** Demographic comparison of study participants and AHPRA psychology workforce data.

Demographics	AHPRA Demographics (*N* = 46,465) %	Total Study Demographics (*N* = 108) %	Chi = Squared (df)	p-value
**Indigeneity**			**233.65 (1)**	**<.001**
Indigenous Australian	0.7	13		
Non-Indigenous	98.3	87		
**Gender**			2.400 (2)	.121
Female	80.5	73.2		
Male	19.5	25.8		
**Age Group**			**18.28 (9)**	.**032**
**<**25	2.6	2.6		
25–29	11.9	12.2		
30–34	13.2	9.6		
35–39	14.0	21.7		
40–44	13.3	7.0		
45–49	11.5	8.7		
50–54	10.1	7.8		
55–59	7.2	3.5		
60–64	6.4	5.2		
65–69	4.7	4.3		
70–74	3.1	0		
75–79	1.5	0.9		
80+	0.5	0.9		
**States of Employment**			**12.895 (5)**	.**024**
QLD	18.2	12.2		
NSW	32	12.5		
VIC	28	21.7		
TAS	1.8	33.9		
SA	4.9	3.5		
ACT	2.5	3.5		
WA	10.5	5.2		
NT	0.6	8.7		
Missing	1.3	2.6		
**Registration Types**			**7.63 (2)**	.**022**
Provisional	16.3	23.5		
Registered	79.8	76.5		
Other	4.0	0		
**Areas of Endorsement**			**65.24 (4)**	**<.001**
Clinical neuropsychology	5.4	21.8		
Clinical psychology	71.5	67.3		
Forensic psychology	4.0	5.5		
Counselling psychology	6.4	5.5		
Community psychology	0.3	0		
Ed and dev psychology	5.5	0		
Health psychology	2.1	0		
Organisational psychology	4.1	0		
Sport and exercise psychology	0.7	0		

The participants’ demographics were compared to the demographic profile constructed by AHPRA to reflect the 2023 psychology workforce. There was an overrepresentation of Aboriginal psychologists (13%) who participated in this study in comparison to the AHPRA workforce population (0.7%). The significant findings highlighted in Table 1 on the age, state of employment, registration type, and area of endorsement are not unexpected given the very large difference between the number of participants, and the number of psychologists within the workforce. Therefore, these findings are not representative of the psychology workforce population.

Of the 108 people who participated in this study, 15 identified as Aboriginal psychologists and were assigned to group 1. A further 73 participants who identified as non-Indigenous psychologists, who indicated they had worked with Aboriginal and Torres Strait Islander clients, were assigned to group 2. The remaining 20 psychologists who identified as non-Indigenous and had not engaged in clinical work with Aboriginal and Torres Strait Islander clients were allocated to group 3. Table 2 displays the participants’ demographics broken down by group as well as a total for the overall sample.

**Table 2.** Comparison of demographic variables for participants separated by group and overall.

	Group 1 Aboriginal psychologists (*n* = 15)		Group 2 Non-Indigenous psychologists, with experience with Indigenous clients (*n* = 73)		Group 3 Non-Indigenous psychologist, with no experience with Indigenous clients (*n* = 20)		Total Sample (*n* = 108)	
	*n*	%	*n*	%	*n*	*%*	*n*	*%*
**Gender**								
Female	13	86.7	59	81.9	17	85	89	83.2
Male	2	13.3	13	18.1	3	15	18	16.8
*Missing*	0		1		0		1	
**Country of birth**								
Australia	15	100	55	76.4	14	70	84	78.5
Overseas	0	0	17	23.6	6	30	23	21.5
*Missing*	0		1		0		1	
**Main Language**								
English	15	100	71	97.3	20	100	106	98.1
Other	0	0	2	2.7	0	0	2	1.9
**Temporary Residency**								
Yes	0	0	1	1.4	1	5	3	2.8
No	15	100	72	98.6	19	95	105	97.2
**Primary Psychology Role**								
Education	1	6.7	7	9.6	5	25	13	12
Direct client services	13	86.7	61	83.6	15	75	89	82.4
Managerial	1	6.7	5	6.8	0	0	6	5.6
Not applicable	0	0	0	0	0	0	0	0
**Secondary Psychology Role**								
Education	5	33.3	18	24.7	8	40	31	28.7
Direct client services	3	20	19	26	1	5	23	21.3
Managerial	1	6.7	5	6.8	2	10	8	7.4
Not applicable	6	40	31	42.5	9	45	46	42.6
**Work across sectors**								
Public	4	26.7	21	30	2	11.1	27	26.2
Private	7	46.7	20	28.6	5	27.8	32	31.1
Both	4	26.7	29	41.4	11	61.1	44	42.7
*Missing*	0		3		2		5	
**States of Employment**								
QLD	3	20	6	8.3	2	10.0	11	10.3
NSW	1	6.7	20	27.8	0	0	21	19.6
VIC	5	33.3	30	41.7	15	75.0	50	46.7
TAS	1	6.7	2	2.8	0	0	3	2.8
SA	0	0	1	1.4	0	0	1	0.9
ACT	0	0	1	1.4	2	10.0	3	2.8
WA	2	13.3	6	8.3	0	0	8	7.5
NT	1 2	6.7 13.3	2 4	2.8 5.6	0 1	0 5	3 7	2.8 6.5
Multiple states								
*Missing*	0		1		0		1	
**Geographical areas of work**								
Metropolitan	7	46.7	50	68.5	18	90	75	69.4
Regional	2	13.3	6	8.2	1	5	9	8.3
Rural	1	6.7	3	4.1	0	0	4	3.7
Remote	0	0	3	4.1	0	0	3	2.8
Multiple	5	33.3	11	15.1	1	5	17	15.7
**Registration or Endorsement type**								
Provisional psychologist	6	40	10	13.7	10	50	26	24.1
Registered psychologist	2	13.3	28	38.4	3	15	33	30.6
Clinical neuropsychology	0	0	10	13.7	0	0	10	9.3
Clinical psychology	7	46.7	18	24.7	6	30	31	28.7
Counselling psychology	0	0	1	1.4	1	5	2	1.9
Double endorsement	0	0	6	8.2	0	0	6	5.6
**Psychology training pathway**								
Doctorate degree	3	20	20	27.8	10	52.6	33	311
Master degree	9	60	31	43.1	7	36.8	47	44.3
5 + 1 Internship	0	0	4	5.6	1	5.3	5	4.7
4 + 2 Internship	3	20	15	20.8	1	5.3	19	17.9
Other	0	0	2	2.8	0	0	2	1.9
Missing	0		1		0		2	

The group comparison shown in Table 3 revealed a significant finding for registration type (*FFH* = 20.43, *p* = .003, *V* = .319). The registration variable explored differences between provisionally registered psychologists and registered psychologists. This indicated that there was a strong significant difference between the groups based on the participant’s type of registration they held. In context, the majority of group 2 participants were registered psychologists, and the majority of group 1, and group 3 participants were provisional psychologists.

**Table 3.** Descriptive statistics for categorical variables between groups using exact hypothesis testing.

	FFH	p-value	*V*	*N*
Gender	.18	1.00	.880	107
Country of birth	5.79	.052	.220	107
Main language	.60	1.00	.095	108
Temporary resident	2.70	.2.45	.127	108
Principle role in psychology	4.29	.317	.153	108
Secondary role in psychology	5.63	.444	.152	108
Work across sectors	5.51	.232	.166	103
State of employment	25.17	.017	.342	107
Geographical areas of work	8.81	.256	.216	108
Registration type	**20.43**	.**003**	.**319**	**108**
Area of endorsement	6.01	.158	.217	80
Psychology training pathway	7.47	.426	.202	106

*Note – **bolded** p-values indicate significance at the p = < 0.005 level*.

No other categorical demographic variables returned a significant finding suggesting no meaningful differences between the three groups on any other categorical demographic variables. However, near significance was obtained for the state of employment, although the majority of participants for each group were working in Victoria. Group 1 also contained a high percentage of participants working in Queensland (20%), while group 2 also had a high percentage of participants working in NSW (28%).

The continuous variables within the study included participants ages, hours spent working in the public sector and the private sector, as well as the number of years they have been practicing as psychologists. The mean and standard deviation for individual groups as well as the overall sample are presented in Table 4 for the continuous variables.

**Table 4.** Comparison of means and standard deviation of continuous variables separated by group and overall sample.

	Group 1 Aboriginal psychologists (*n* = 15)		Group 2 Non-Indigenous psychologists, with experience with Indigenous clients (*n* = 73)		Group 3 Non-Indigenous psychologist, with no experience with Indigenous clients (*n* = 20)		Total Sample (*n* = 108)	
	*M*	*SD*	*M*	*SD*	*M*	*SD*	*M*	*SD*
**Age**								
Range in years	24–65		22–83		23–60		22–83	
Average in years	42	12.8	40.1	13.1	41.3	10.5	40.7	12.8
**Sector work (hours)**								
Public	27.3	11.5	28.4	10.3	23.2	14.4	27.7	10.8
Private	23.4	14.7	20.6	11.8	20.7	9.7	20.7	11.9
**Years worked as psychologist**	8.3	7.3	12.8	11.1	10.9	5.1	10.9	10.1

Making no assumptions about the distributions, a Kruskal-Wallis test revealed a statistically significant difference in the number of years the participants had worked in psychology between the three groups, (χ^2^(2) = 8.705, *p* = .013). Pairwise comparisons on the age and years worked in psychology variables indicated a significant difference between group 3 and group 2 respectively (χ^2^ = 18.731, *p* = .018; χ^2^ = 22.680, *p* = .004). The Kruskal-Wallis showed no significant group differences between age and hours worked by participants across the public and private sectors.

Outside the demographic information collected, all participants regardless of group allocation were asked how the clinic or organisation that they work for identify if their clients are Aboriginal or Torres Strait Islander. This was collected through an open-ended question with a box for free text in the survey that participants completed. Six main themes emerged from the responses, these are displayed in Table 5.

**Table 5.** Psychology clinics and organisations methods to identify Aboriginal and Torres Strait Islander clients.

Main Themes	%	Quote Example
Intake or administration	36.6	“A screening question is asked at intake”
Self-reported	20.8	“Clients self-identify”
File or referral	13.9	“Via medical records”
Initial assessment	15.8	“I ask about all my client’s cultural identity in my initial session”.
Network connections	5.9	“Word of mouth, family-connections”
Unsure or do not collect	6.9	“They don’t”

*Note: n = 101 missing = 7*

Table 5 depicts the six main themes that emerged from undertaking content analysis on the open-ended questions aimed to explore how psychology clinics and organisations identify their Aboriginal and Torres Strait Islander clients. The findings state that 36.6% of participants suggested their clients’ identities are captured using intake or admin forms, 20.8% of participants indicated they rely on the clients to self-report their identity during sessions, while 15.8% of participants suggested they ask clients as part of their initial assessment process. Less than 1 in 7 participants (13.9%) suggested they rely on the referral information or the clients’ files that is compiled usually as part of the system they work in (e.g., justice, hospital). Another 6.9% of participants indicated they do not collect this information, or are unsure of their organisation’s practice in collecting this information. While 5.9% of participants, majority Aboriginal, reported that their services are sought out due to their identity, or community connections.

**Figure 1.**
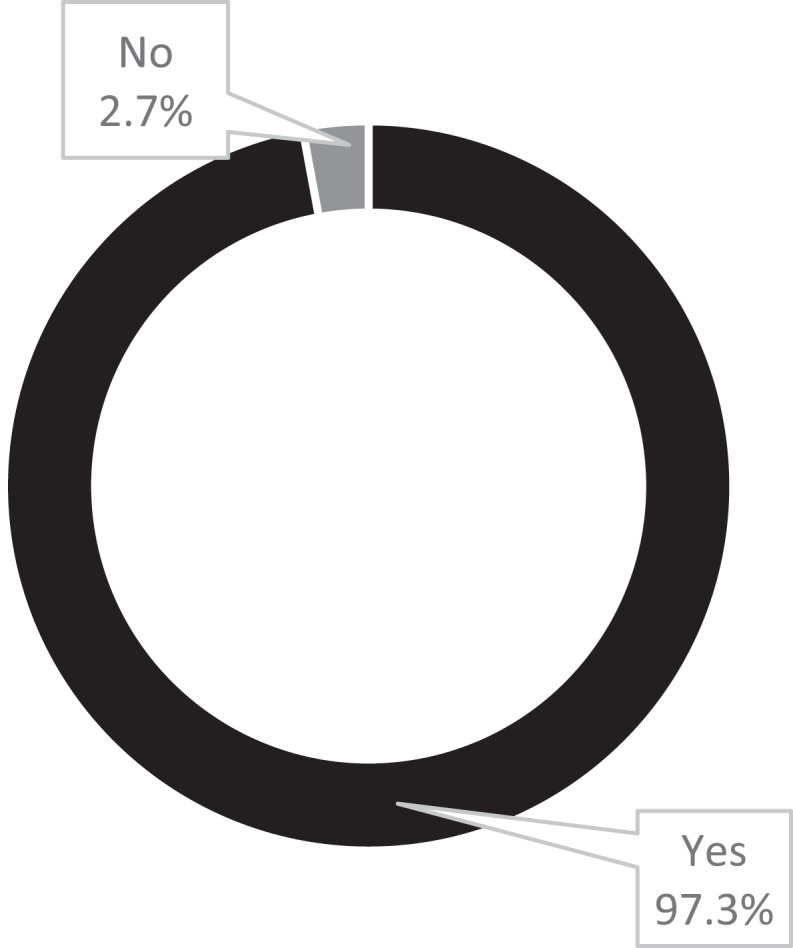
Participants’ perspectives of the need for psychological practice adjustments when working with Aboriginal and Torres Strait Islander clients.

Understanding the identity of clients who present for psychological services is believed to be crucial to inform the choices for intervention and practices that psychologists undertake. To understand psychologist’s beliefs about the need to adjust their practice when working with Aboriginal and Torres Strait Islander clients, all participants were asked if they believed practice adjustments were necessary when working with Aboriginal and Torres Strait Islander clients. Figure 1 displays these results.

*n = 113, missing = 2*

Figure 1 highlights that 97.3% of participants believe psychologists need to change their clinical practice when working with Aboriginal and Torres Strait Islander clients. Importantly, 100% of the Aboriginal participants agreed that psychological practice adjustments are needed when engaging with Aboriginal and Torres Strait Islander clients. Of the 2.7% that reported “no” in Figure 1 (*n* = 3), two participants justified their answers.

“If they are good clinicians”. (Participant A, Group 2)

I treat all people like “people”. Aboriginal identity is no more unique than any other cultural subset. (Participant B, Group 2)

**Figure 2.**
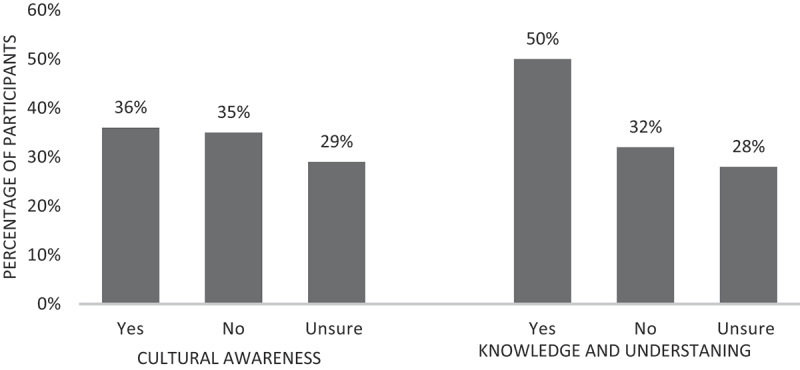
Non-indigenous participants reported ability to provide competent psychological therapy to Aboriginal and Torres Strait Islander clients based on current cultural knowledge, understanding, and awareness.

The majority of participants indicated the need for psychologists to adjust their practice when working with Aboriginal and Torres Strait Islander clients. However, 64% of non-Indigenous participants reported that they did not, or were unsure, if they had enough cultural awareness to provide competent psychological therapy to Aboriginal and Torres Strait Islander clients. Further, participants were asked if they thought they had enough knowledge and understanding about the Aboriginal and Torres Strait Islander culture to provide competent psychological therapy to a client who identified within the culture. Fifty percent of participants indicated they did not, or were unsure, if they had enough knowledge and understanding to provide competent psychological therapy to a client. These findings are depicted in Figure 2.

All participants allocated to either group 1 or 2 were asked about the challenges they have experienced when working with Aboriginal and Torres Strait Islander clients. Aboriginal participant’s responses in group 1 were combined into 3 main themes with 100% of the participants referencing at least one systematic and/or organisational barrier in their response. Four main themes emerged from non-Indigenous psychologists who have experience working with Aboriginal and Torres Strait Islander clients in group 2. The main themes of *Complex Presentation*, and, *Systemic and Organisational Barriers* were mentioned in participants responses the most with 52.5% and 54% of participants referencing a challenge within these themes respectively. Aboriginal psychologists’ main theme of *Ethical Implications* (33%) was not shared by non-Indigenous psychologists, although some reported ethical challenges experienced were shared and combined into other main themes. Contrastingly, only non-Indigenous psychologists reported the challenges that encompassed the main themes *Under Resourced* (29%) and *Lack of Knowledge* (25.5%) apart from one challenge that participants indicated the need for more Aboriginal and Torres Strait Islander psychologists or health professionals. Table 6 displays the participant’s reported challenges.

**Table 6.** Challenges experienced by psychologists when working with Aboriginal and Torres Strait Islander clients.

	Main Themes	Percentage*	Challenges Experienced
Aboriginal psychologists (*n* = 15)	Ethical Implications	33%	Initial contact or **engagement** Dual relationships Informed consent Confidentiality **Interface of cultural and ethical practices not aligning**
	**Complex Presentation**	40%	Complex multilayered presentations **Complex environmental factors** High levels of intergenerational trauma Client experience resonates with personal experience.
	**Systemic or Organisational Barriers**	100%	**Lack of personal/government funding to continue.** Layered systemic disadvantage **Negative past experiences in the health system** Lack of Indigenous knowledge requirements by regulatory bodies **Client mistrust of systems** **Lack of Aboriginal psychologists and staff in the systems** Lack of culturally safe mental health services
Non-Indigenous psychologists (*n* = 59 missing = 14)	**Complex Presentation**	52.5%	More neurodevelopmental challenges than non-Indigenous **Complex trauma presentations** Historical impacts Getting a thorough history from clients Parental involvement with younger clients Difficulty engaging clients Difficulty interpreting non-verbal cues Diversity within acculturation Comorbid health issues Family and community obligations impacting engagement and progress Rapport building Absence of basic human rights
	**Systemic or Organisational Barriers**	54%	**Clients deep mistrust of systems** Inadequate diagnostic procedures (overdiagnosed) **Interface of culture and ethical practice** Receiving conflicting best practice training Difficulty attending appointments Lack of flexibility in organisation policies Reduced mental health literacy Working appropriately within multi-disciplinary teams **Restrictions imposed by the Medicare model** **Interface of implementing best practice within funding models** Strict and unethical justice system policies and procedures **Negative past experiences in the health system** **Service engagement**
	Under Resourced	29%	Less access to services Less opportunity Less resources Less evidence-based therapies/interventions Less services for referral and support Limited interpreter services Additional time burden on psychologists to educate themselves and find appropriate resources **Lack of Indigenous AHPRA professionals** Lack of appropriate supervisors
	Lack of Knowledge	25.5%	Not understanding cultural values/traditions Not understanding the connection to Country Fear of perpetuation of unintentional burden or harm Not familiar with culture-bound syndromes Uncertainty of how to gather information appropriately Unsure of correct language and terminology Lack of confidence Inadequate university education/training for psychologists Impact of Aboriginality in presentation Applying a Cultural lens Feeling ill-equipped to respond to revelations so far removed from professional knowledge or experience. Language barriers Being conscious of personal bias, privilege, and assumptions

*Note – There was no limit on how many themes participants could address in response to this open-ended question, therefore, the percentage will not equal 100, and rather reflects the percentage of participants who referenced the theme in their open-ended response.

**Bolded** responses represent a commonly report theme across both groups.

Both Aboriginal and non-Indigenous psychologists in groups 1 and 2 were asked about the psychological practices they have found to be successful when working with Aboriginal and Torres Strait Islander clients. Table 7 displays these results.

**Table 7.** Thematic comparison of suggested successful psychological practice adjustments when working with Aboriginal and Torres Strait Islander clients between Aboriginal and non-Indigenous psychologists.

Participant Group	Theme	Percentage*	Suggested Successful Practice Adjustments
Aboriginal psychologists (*n* = 15)	Shared Knowledge	33%	Understanding how culture shapes Aboriginal and Torres Strait Islander peoples Identity **Trauma** **Systemic barriers** **Historical barriers**
	Indigenous Worldviews	86%	Applying: **Strengths-based approach,** **Working holistically,** **Incorporating the SEWB framework** **Ensuring self-determination,** **Working collaboratively with family, community, or services, when necessary,** **Incorporating a trauma-informed approach**.
			Removing (when appropriate): **Diagnostic lens** Westernised epistemologies, Scientist-practitioner model when appropriate.
	Community Focused	20%	Incorporating and addressing: Community healing **Local supports** Social determinants of health
	**Therapeutic Process**	73%	Participants suggested: **Need to move more slowly.** **Build deeper trust and a secure therapeutic relationship.** **Increased self-disclosures**. Establishing shared community connections **Carefully exploring how the community relationships differ from the therapeutic relationships**. Take a horizontal, **collaborative, and informal approach**. Address diversity within the Aboriginal and Torres Strait Islander communities to understand acculturation. **Sessions outside in nature.** **Daddiri (Deep listening)** Make information simple and manageable. Use visual cues. **Undertake cultural mapping.** **Considering the language used to communicate psychological information.** **Utilising narrative** and storytelling approaches. **Walking alongside the clients**, meeting them where they are at.
Non-Indigenous psychologists (*n* = 57, missing = 16)	Personal Characteristics	30%	Important Characteristics: Empathy Honesty Humility Openness Curiosity Advocacy Flexibility Willingness to learn Respect Non-Judgemental Genuine Integrity Humour
	Interventions and Approaches	58%	Successful outcomes after implementing: Systemic/Group therapies **Narrative therapy** Art and play therapy **Using SEWB frameworks** Less formal and structured sessions Reflexivity to changing needs of clients **Cultural mapping** **Daddiri (deep listening)** A mindful state **Trauma-informed care** Acceptance Client-centred approach Collaborative formulation with client and wider community of appropriate
	**Therapeutic Processes**	87%	Participants suggested: **Increase self-determination** **Increased self-disclosures** **Walking alongside the clients** **Holistic, bigger picture approach** **Strengths-based approach** **Moving at a slower pace in session** Establish and explore common ground more **Remove diagnostic lens** Yarning style communication Less reflective feedback Extended Information gathering Extended time spent rapport building **Explicit communication of roles** Explicitly addressing confidentiality and consent Open invitation for community to attend sessions if the client grants permission Invite feedback and corrections from client Explaining reasoning behind questions asked **Adjust language used** Explore client’s past experiences with psychologists Sitting next to the client as opposed to opposite Consider cultural norms around eye contact Increased awareness of psychologist’s non-verbal cues
	Knowledge and Professional Development	56%	Understanding, exploring, and/or addressing: Connection to culture **Historical and continuing impact of historical events** Underlying assumptions Generalised information in cultural competency training Increased exposure to grief and loss Placing caveats in assessment reports addressing scientific and ethical implications The use of invalid psychometric assessment tools. Unlearning Westernised education and relearning decolonised practice methods Seek Aboriginal supervision, cultural consultants, or Aboriginal colleagues in the workplace for guidance on cultural aspects of clients Professional development opportunities Reduce power imbalance by “dressing down” **Increase accessibility** (e.g., provide taxi vouchers) Aboriginal and Torres Strait Islander worldviews Importance of Country **Cultural identities are individual and diverse**
	Cultural and Environmental Considerations	35%	Consider: Flexible locations for sessions (e.g., outside) Accounting for spirituality Connecting with community elders **Work collaboratively with Aboriginal services** Cultural engagement as a protective factor Display Aboriginal art in the office Aiding connection to community for clients who have relocated Offer home visits Engage with community outside of individual clients to deepen knowledge and strengthen personal connections

*Note – There was no limit on how many themes participants could address in response to this open-ended question, therefore, the percentage will not equal 100, and rather reflects the percentage of participants who referenced the theme in their open-ended response.

**Bolded** responses represent a commonly report theme or suggestion across both Aboriginal and non-Indigenous psychologist groups.

Table 7 displays the participants’ suggested successful psychological practice adjustments when working with Aboriginal and Torres Strait Islander clients. Aboriginal participants responses were coded in four main themes *(Shared Knowledge, Indigenous Worldviews, Community Focused, Therapeutic Process*), while non-Indigenous participants responses were coded in five main themes *(Personal Characteristics, Interventions and Approaches, Therapeutic Processes, Knowledge and Professional Development, Cultural and Environmental Considerations*). Unsurprisingly, considering the topic being explored in this paper, the Therapeutic Processes theme was shared across both groups. Similarly, both Aboriginal and non-Indigenous psychologists suggested successful practice adjustments that aligned with the Therapeutic Processes theme more so than any other theme. No other themes were shared, however, there were some similarities between the remaining main themes presented between the two groups such as knowledge and culture.

The practice adjustments that were mentioned across both groups were bolded for easy identification. There was the most overlap between the two groups within the Therapeutic processes (group 1 and 2), Indigenous worldviews (group 1), and Intervention/approach (group 2). The practice adjustments endorsed by both groups included deep listening, narrative therapy, self-determination, being trauma-informed, practicing holistically, taking a strengths-based approach, moving slowly in session, and removing the diagnostic lens.

Some successful practice adjustment suggestions between groups contradicted each other. Examples of this include non-Indigenous psychologists suggesting a list of personal characteristics such as “empathy”, “curiosity”, and “openness” to be a successful practice adjustment when working with Aboriginal and Torres Strait Islander clients, which was inconsistent with Aboriginal psychologists’ suggestions. Further, Aboriginal psychologists placed great emphasis on undertaking a community approach to healing. For the differences between groups, it is important to privilege Aboriginal voices and suggestions as the custodians of the culture and lived experiences within it ensure Aboriginal people are the best sources of knowledge when it comes to understanding, exploring, and meeting our own needs.

To further explore the differences between groups, the participants were also asked what practice adjustments they have not found to be successful when working with Aboriginal and Torres Strait Islander clients. The results are displayed in Table 8.

**Table 8.** Thematic comparison of experience of unsuccessful psychological practice adjustments when working with Aboriginal and Torres Strait Islander clients between Aboriginal and non-Indigenous psychologists.

Participant Group	Theme	Percentage*	Experience of Unsuccessful Practice Adjustments
Aboriginal psychologists (*n* = 15)	**Therapeutic Process**	40%	**Rushing** **Not spending enough time building rapport** Exploring Emotions too quickly Being too direct Sterile therapeutic environments
	**Intervention or Approaches**	26.5%	Adjustments to cultural practice when clients are seeking a diagnosis. A pedagogical approach Surface level changes to Western ways of working
	**Systemic or Organisational Barriers**	20%	Inflexible systems and policies to provide best practice.
Non-Indigenous psychologists (*n* = 51 missing = 22)	**Therapeutic process**	39%	Not adjusting for cultural differences Speaking too much Spotlighting during training and education contexts Communicating poorly, increasing client’s shame Blank face acceptance Ignoring the power imbalance **Being impatience** Waiting for clients to attend the clinic Being too fearful to attempt adjustments Not checking in with clients for clarity **Not building enough rapport before progressing** Not giving clients choices in their care Directly explaining Indigenous frameworks (questions work better) Being too conservative Confined/small counselling spaces.
	Identity	23.5%	Being ingenuine as the psychologist Generalised group assumptions based on identity Not addressing psychologist’s white privilege Imposing personal views and stereotypes
	**Systemic or Organisational Barriers**	25.5%	Referring directly to Indigenous services once identified Not addressing social/systemic issues Formal paperwork Accepting and applying DSM definitions or criteria Assuming referral reason is the only challenge they are experiencing Differing perceptions of confidentiality GP referrals for medical intervention without client having a relationship with the GP Using inappropriate resources Not understanding the services available to clients Not understanding roles of people involved with clients Emphasising organisational policy and procedure Clients having to educate psychologists about culture
	**Interventions or Approaches**	12%	Short-term therapies addressing symptoms. Using CBT Applying Westernised attachment theories Using a deficit approach and language

*Note – There was no limit on how many themes participants could address in response to this open-ended question, therefore, the percentage will not equal 100, and rather reflects the percentage of participants who referenced the theme in their open-ended response.

**Bolded** responses represent a commonly reported theme by both groups.

Table 8 illustrates the participants’ experiences of unsuccessful practices that they have experienced working with Aboriginal and Torres Strait Islander clients. Aboriginal psychologists’ suggestions were compiled into three main themes (*Therapeutic process, Intervention of Approaches*, and, *Systematic or Organisational Barriers*), with 26.5% of participants suggesting they had not experienced any unsuccessful practice adjustments. Non-Indigenous psychologists’ reports were combined into four main themes. With 17.5% of participants in group 2 also reported they had not or were unsure if they experienced any unsuccessful practice adjustments. Interestingly, these four themes were the same as the three main themes that emerged from Aboriginal psychologists’ findings with the addition of an *Identity* theme. However, despite the similarities in main themes, the underlying unsuccessful practices suggested by the participants do not overlap as much as previously experienced in other questions in this research.

## Discussion

The paper provides insights into successful, and unsuccessful ways psychologists have tried to embed cultural responsivity in clinical practice with Aboriginal and Torres Strait Islander clients. More specifically, this research compared Aboriginal and non-Indigenous psychologists’ experiences and suggestions around what they thought was challenging, successful, and not successful when engaging in psychological practice with Aboriginal and Torres Strait Islander clients. Therefore, this research makes a significant contribution to the small body of literature available to guide non-Indigenous psychologists towards more culturally responsive psychological practice.

Collectively the findings revealed that the majority of participants believe psychological practice adjustments are necessary when providing psychological support to Aboriginal and Torres Strait Islander clients. However, the practice adjustments suggested by participants were diverse and at times contradicted one another between groups. This may reflect the diversity within the Aboriginal and Torres Strait Islander communities. Additionally, it may also be underpinned by the suggested lack of Indigenous cultural education and understanding by non-Indigenous psychologists.

Notable differences between the Aboriginal psychologists and non-Indigenous psychologists’ reports exist in the findings. Given the inherent cultural knowledge held by Aboriginal psychologists, the non-Indigenous psychologists having a commonly occurring additional main theme such as “*Lack of Knowledge”* was a clear point of difference. The lack of cultural training for psychologists makes this difference unsurprising (Dudgeon et al., 2014; Gee et al., 2014; Geerlings et al., 2018; Mcconnochie et al., 2012; Mullins & Khawaja, 2018; Westerman & Dear, 2023). To address the challenges and bridge the gaps between the shortcomings evidenced in the education systems the onus is placed on the psychologists to upskill. Despite some non-Indigenous psychologists displaying explicit and implicit racial bias in their responses (e.g., othering language), it was accompanied by the majority of participants in this group recognising their knowledge and cultural gaps, as well as an intrinsic desire to improve their clinical and cultural responsiveness. Kilcullen et al. (2018) suggested engaging in critical reflection to understand individual limitations as a psychologist is important to improving clinical practices. Further, Westerman (2010) advocated for the use of cultural consultants to guide non-Indigenous psychologists through complex cultural presentations and to improve culturally responsive engagement styles, and hopefully increase service uptake by Aboriginal and Torres Strait Islander clients.

The themes from the research mostly align with the body of literature that exists exploring this topic. The challenges experienced by non-Indigenous psychologists highlighted in previous literature, such as the need to understand historical and current influences of wellbeing, cultural norms, worldviews, communities, specific contexts, misdiagnosis, and service engagement (Clark & Hirvonen, 2022; Dudgeon et al., 2014; Dudgeon & Walker, 2015; Gee et al., 2014; Westerman, 2004, 2010; Westerman & Dear, 2023) were reinforced several times throughout this research. These findings further add to the knowledge about the challenges that Aboriginal psychologists face when working with Aboriginal and Torres Strait Islander clients. Highlighted are the problematic systemic and organisational barriers that exist to provide culturally responsive services to Aboriginal and Torres Strait Islander peoples as highlighted by the participants in this research.

Specific psychological interventions that were suggested by participants are featured throughout the findings of this study. In line with Ponturo and Kilcullen’s findings (2021), narrative therapy was strongly reinforced by both Aboriginal and non-Indigenous psychologists to be a culturally responsive and useful intervention when working with Aboriginal and Torres Strait Islander peoples. Also similar to Ponturo and Kilcullen’s (2021) findings, only non-Indigenous psychologists mentioned the use of acceptance-based therapies. Ponturo and Kilcullen’s (2021) found CBT to be applicable cross-culturally. However, some non-Indigenous psychologists in this study suggested CBT to be an unsuccessful practice adjustment. These findings may be explained by Dudgeon and Kelly’s (2014), commentary supporting the need to use CBT in conjunction with other interventions and worldviews when working with Aboriginal and Torres Strait Islander clients. Future research should explore the utility of CBT within an Aboriginal and Torres Strait Islander context further to understand the impacts, as well as explore ways in which CBT can be adapted to be culturally informed and responsive.

Psychological practice adjustments such as, applying Indigenous frameworks, worldviews, community-based approaches, relational, holistic, strengths, and values-based approaches are commonly and consistently reported in past literature to be useful tools when working with Aboriginal and Torres Strait Islander clients (Bullen et al., 2023; Clark & Hirvonen, 2022; Coombs, 2023; Dawson et al., 2023; Dudgeon et al., 2014; Gee et al., 2014; Gee et al., 2023; Kilcullen et al., 2016; Krakouer et al., 2022; Mullins & Khawaja, 2018; Murrup‐Stewart et al., 2019; J. O’connor et al., 2015; Rhodes & Langtiw, 2018; Westerman 2004, 2008, 2010; Westerman & Sheridan, 2020). Although, these concepts can still at times remain ambiguous about how to be applied in practice. For example, research suggests applying a strengths-based approach when working with Aboriginal and Torres Strait Islander clients to be more effective in enhancing SEWB (Kilcullen et al., 2018). However, there is less research that explores the practical application of a strengths-based approach. Further research should explore how to appropriately apply these practices when working with Aboriginal and Torres Strait Islander clients.

More specifically, Mullins and Khawaja’s (2018) findings from their study were mostly supported by this research. Practices such as engaging community, being flexible, honest, transparent, and empowering clients, having an understanding of intergenerational trauma, using culturally validated assessment tools, and culturally sensitive communication strategies were all mentioned by both Aboriginal and non-Indigenous psychologists. The findings are strengthened by Aboriginal psychologists reporting the practices to be successful to use within an Aboriginal and Torres Strait Islander context. This paper, therefore, addressed Mullins and Khawaja’s (2018) study limitation by including Aboriginal psychologist’s voices.

### Limitations

Firstly, there were no Torres Strait Islander psychologists who participated in this study. Therefore, the applicability of these findings without specific expertise and insights from Torres Strait Islander psychologists should be done with appropriate caution. This limitation could act as a point for future research. Additionally, the parameters of group allocation should have been considered further. For example, a participant in group 2 had previously engaged an Aboriginal client in only one session. Therefore, their contribution to the data was limited, and would likely have made a more meaningful contribution if allocated to group 3. While no limit currently exists as to how much is “enough” experience with Aboriginal and Torres Strait Islander clients, further consideration, and more relevant allocation alignment with skills and experience could have been made. Further to the limitation based on group allocation, a significant difference was calculated for the demographic variables of registration type. This is due to the high number of provisional psychologists that comprised groups 1 (40%) and 3 (50%). While this significant difference is not surprising given the basis for group allocation, it should be noted that provisional psychologists are still considered to be in training. Further research could address these considerations and replicate this study to explore these research questions with experienced psychologists to add more validity to the findings.

Successful and unsuccessful practice adjustments could have been more clearly defined when requesting participants to draw upon their experiences. The lack of defined indicators of success introduced error through the participants subjective interpretation of what they considered successful and unsuccessful. Evidenced in the responses from non-Indigenous participants was conscious and unconscious bias, discrimination, and a suggested lack of understanding around culturally responsive practice, therefore the individual participant perception of what they viewed as successful, may be problematic and align with Western ideologies. This provides a further rationale for the need for the current research and will be explored in greater depth in the following associated paper. Consequently, the suggestions from the non-Indigenous cohort should be further considered before implementation if they do not overlap with the Aboriginal participant’s suggestions or are not already validated within the existing body of literature. Future research could address this study limitation by taking the findings from the non-Indigenous participants that do not overlap with the Aboriginal participant’s suggestion to the Aboriginal and Torres Strait Islander community members for further consideration and validation.

### Implications

A clear strength of the study is depicted in the demographic makeup of the participants. There is a strong Aboriginal presence in this study (13%) in comparison to the 0.7% of the psychological workforce in Australia. This displays strong support for the need for this research from the Aboriginal community. Further, in line with privileging Aboriginal voices, the findings from this study should be considered deeply as it represents one of the most significant reports of Aboriginal psychologist voices in published literature. The significant findings highlighted in Table 1 on the age, state of employment, registration type, and area of endorsement are not unexpected given the very large difference between the number of participants. Therefore, while these findings are not an accurate representation of the psychology workforce they still offer deep insights into how non-Indigenous psychologists can learn from the Aboriginal psychologists to work towards improving their psychological practice.

The current findings may be important to influence policy and practice. A clear cry for policy reform in systems, university training programmes, clinics, and organisations has been consistently reinforced throughout this paper. Aboriginal and Torres Strait Islander peoples are at risk of being misdiagnosed at higher rates due to the lack of culturally validated psychometric tools, as well as the clinician’s inability to consider the culture and the context of the individuals (Clark & Hirvonen, 2022; Dudgeon et al., 2014; Dudgeon & Walker, 2015; Gee et al., 2014; Westerman, 2004, 2010; Westerman & Dear, 2023).

## Conclusion

The current paper’s findings reiterate the similarities and differences between Aboriginal and non-Indigenous psychologists’ approaches to working with Aboriginal and Torres Strait Islander clients. To address the disparities and improve Aboriginal and Torres Strait Islander SEWB current non-Indigenous psychologists should engage with professional development, cultural supervision, and critical reflection outside of the Westernised lens. Further, to better prepare the future psychological workforce, higher education systems need to be adequately funded to decolonise the psychology curriculum and upskill educators.

## Data Availability

The participants of this study did not give written consent for their data to be shared publicly, so due to the sensitive nature of the research supporting data is not available.
